# Mini-Review on Lipofuscin and Aging: Focusing on The Molecular Interface, The Biological Recycling Mechanism, Oxidative Stress, and The Gut-Brain Axis Functionality

**DOI:** 10.3390/medicina56110626

**Published:** 2020-11-19

**Authors:** Ovidiu-Dumitru Ilie, Alin Ciobica, Sorin Riga, Nitasha Dhunna, Jack McKenna, Ioannis Mavroudis, Bogdan Doroftei, Adela-Magdalena Ciobanu, Dan Riga

**Affiliations:** 1 Department of Biology, Faculty of Biology, “Alexandru Ioan Cuza” University, Carol I Avenue, no 20A, 700505 Iasi, Romania; 2 Academy of Romanian Scientists, Splaiul Independentei, no. 54, sector 5, 050094 Bucharest, Romania; sorin.riga@gmail.com (S.R.); d_s_riga@yahoo.com (D.R.); 3 Mid Yorkshire Hospitals NHS Trust, Pinderfields Hospital, Wakefield WF1 4DG, UK; nitasha.dhunna@doctors.org.uk; 4 York Hospital, Wigginton road Clifton, York YO31 8HE, UK; jackmckenna@doctors.org.uk; 5 Leeds Teaching Hospitals NHS Trust, Great George St, Leeds LS1 3EX, UK; i.mavroudis@nhs.net; 6 Laboratory of Neuropathology and Electron Microscopy, School of Medicine, Aristotle University of Thessaloniki, 541 24 Thessaloniki, Greece; 7 Faculty of Medicine, University of Medicine and Pharmacy “Grigore T. Popa”, University Street, no 16, 700115 Iasi, Romania; bogdandoroftei@gmail.com; 8 Discipline of Psychiatry, Faculty of Medicine, “Carol Davila” University of Medicine and Pharmacy, Dionisie Lupu Street, no 37, 020021 Bucharest, Romania; adela.ciobanu@yahoo.com

**Keywords:** aging, lipofuscin, molecular biology, oxidative stress, autophagy, gut–brain axis

## Abstract

Intra-lysosomal accumulation of the autofluorescent “residue” known as lipofuscin, which is found within postmitotic cells, remains controversial. Although it was considered a harmless hallmark of aging, its presence is detrimental as it continually accumulates. The latest evidence highlighted that lipofuscin strongly correlates with the excessive production of reactive oxygen species; however, despite this, lipofuscin cannot be removed by the biological recycling mechanisms. The antagonistic effects exerted at the DNA level culminate in a dysregulation of the cell cycle, by inducing a loss of the entire internal environment and abnormal gene(s) expression. Additionally, it appears that a crucial role in the production of reactive oxygen species can be attributed to gut microbiota, due to their ability to shape our behavior and neurodevelopment through their maintenance of the central nervous system.

## 1. A Retrospective View of Our Current Knowledge of the “Age Pigment”

Aging is an irreversible process characterized by a progressive dysregulation of homeostasis, inherent to life’s finite cycle. The influence exerted by this phenomenon leads to a series of molecular abnormalities [1]. Fortunately, discoveries were made over decades, concerning the involvement of so-called “wear-and-tear”. Therefore, the advent of biomedicine has become imperative [2].

Most studies found in the current literature aimed to establish a boundary between the origin of lipofuscin compared with that of ceroid [3]. These two structures are the main markers of brain vulnerability, oxidative stress, and senescence/senility related pathologies [4]. Even if they influence a similar spectrum of diseases, a clear differentiation was needed [5].

Characteristic neuropathological aging profiles are influenced by lipopigment concentration, which generates a cascade of negative events at a subcellular level. As our group previously demonstrated, these specific and associated negative consequences of lipopigment accumulation have multiple detrimental effects on neuron and glia homeostasis, from neuronal function to central nervous system (CNS) physiology [4].

We also revealed that common mechanisms in the mammalian brain in distress, through aging and neurodegeneration, are mainly through hypoanabolism, or a reduction in RNA and protein synthesis, coupled with hypercatabolism, leading to an increase in oxidative stress—lipid peroxidation protein insolubilizing with waste-trash products accumulating, such as ceroid lipofuscin [6,7,8]. In contrast, lysis and elimination of the neuronal lipopigments are some of the main mechanisms for the re-establishment of metabolic, cellular, and tissue homeostasis, suggesting an antioxidative stress, anti-aging, and regenerative therapy [9].

Multicellular organisms are composed of heterogeneous mechanisms throughout multiple systems of organs. Thereby, organismal senescence is depicted by a gradual diminution of both physiology and functions of cells, with the cause–effect having high repercussions upon overall structural integrity [10].

Despite that lipofuscin accumulates in all somatic cells, its accumulation is blocked by an integrated mechanism to all pro- and eukaryotic cells. Therefore, the cell cycle prevents the accumulation of lipofuscin, and although this imperatively occurs at differing rates [9], non-dividing ones [11] are the most susceptible. As highly oxidized crosslink aggregated “wastes”, lipofuscin possesses a structure that is as unique as it is controversial.

Biological recycling mechanisms are unable to act efficiently [12], and although an undegradable property defines lipofuscin, it seems that mitochondria play a pivotal role [13]. These aspects may culminate in increasing the risk of destabilizing homeostasis, which is why, in the next sections, we detail the underlying interconnections between them.

## 2. Does Lipofuscin Really Possess a Branched Antagonistic Activity on Homeostasis?

For this section, we searched the databases based on keywords that include “lipofuscin”, and to our surprise, we identified only a few after 2010. The most recent article published is that of Kakimoto et al. [14], in 2019. Alongside their collaborators, they collected seventy-six postmortem cardiac samples from patients ranging between 20 and 97 years. Following a series of investigations, they demonstrated that lipofuscin accumulation is positively associated with chronological aging, but independently with other parameters, such as body index and weight, cause of death, or levels of brain natriuretic peptide (BNP). They concluded that lipofuscin is not involved in human cardiac pathologies, but rather a consequence of aging.

It has also been demonstrated that lipofuscin presence is not only restricted to humans, but to other mammals, as well. Aiming to investigate the expression of both autophagy and amyloid precursor protein (APP) markers in the brains of aged bovine, De Biase et al. [15] collected thirty samples and divided them into two groups, based on age. According to their results, intraneuronal accumulation of lipofuscin was the most consistent finding, with this information adding to that of Kakimoto where they reveal an accumulation in the perinuclear zone. Based on their results, immunoreaction to APP, Beclin-1 (Atg6 orthologue) and LC3 (microtubule-associated protein 1 light chain 3) proved to be either weak or absent. They concluded that autophagy is significantly impaired in contrast to young specimens, reflected by an APP deposition dependent by age.

It has been also documented on rats that zinc deficiency promoted an accumulation of waste-trash products retinal pigment epithelium (RPE), concomitantly with lipofuscin-loaded macrophages in the choroids followed by the presence of such cells at Bruch’s membrane [16]. While Julien revealed that a zinc-based diet could exert beneficial effects on adult Long Evans (LE) rats, it was demonstrated this year, by Wang et al. [17], through transmission electron microscope, that myocardial tissues of mice possess the ability to eliminate unnecessary bio-products into the associated bloodstream.

However, within the established interval (2010–2020), we identified just one study in which there were over one hundred samples collected. This is the study of Nozynski et al. [18], where the authors collected one hundred and thirty-six hearts explanted. It should be mentioned that these samples were collected from patients that suffered from various metabolic and vascular disorders or donors as control group. Congruent with the aforementioned, lipofuscin was present inside advanced glycation end product (AGE) of all studied groups. Considering that frequencies did not differ significantly, they claim that lipofuscin could belong to AGE deposits.

Considering that lipids represent the main underlying elements behind lipofuscin formation, an impairment of lipid metabolism proved to be the starting point of lipofuscin accumulation and synthesis. Contrary to expectations, the human body is not a perfect mechanism, and this is all the more so as aging promotes a series of crucial changes. One example in this context is represented by the gradual disturbance of cellular proteostasis. More precisely, there is an impediment to the degradation of the folded proteins and thus results in complexes formed with other perinuclear/centrosomal-proximal proteins with the risk to aggregate into aggresomes [19,20,21,22,23,24].

Theoretically, lysosomal degradation should be rapid and effective, but, in reality, the process is far more complex. During macroautophagy dedicated to lysosomal uptake, degradation seems to occur alongside iron-catalyzed peroxidation, which, in turn, leads to lipofuscin accumulation [25], which is a process marked by a concentration of lipofuscin in lysosomes and cytosol [26].

In a recent review of Höhn et al. [27], the authors offer a systemic mechanical point of view regarding lipofuscin interaction and effects on homeostasis. They even discuss the crosstalk between degradation pathways and suggest that the ubiquitin-proteasome system (UPS) and autophagy are negatively influenced by lipofuscin. While proteasome degrades short half-life proteins in a personalized manner, autophagy, on the other hand, is attributed to cleansing long half-life proteins. Even though was initially thought that UPS and autophagy act individual, recent data indicate that these systems could actually be intertwined by sharing particular substrates and regulatory particles [28,29]. Intriguingly, proteasomal inhibition induces autophagy, but it is a one-way circuit [30], being speculated that ubiquitin coordinates the catabolism of both UPS and autophagy.

Korolchuk et al. [31] discuss how the proteasomal system preferentially degrades K48-linked polyubiquitin chains, while K63-linked chains are modified or monoubiquitinated. Kirkin et al. [32] took this topic further and showed that these linkers possess similar components such as SQSTM1/p62, NBR1, HDAC6, and Alfy, with the last one being proposed as a linker between UPS and autophagy. There is an increased interest in the current literature regarding the role of p62 as a substrate through which are formed homo-oligomers and how ubiquitinated proteins are recruited via its c-terminal UBA [32,33].

As has been already extensively documented by Höhn and Grune [26], proteasome not only exists in different forms, but its biological activity is dependent and modulated by a series of regulators (20S known as the core, 19S regulators, and 26S), with the last one resulting from a merger between 20S and 19S. Individually, the 20Ss fulfill crucial roles, such as oxidized protein degradation [34,35,36,37], similar to other substrates [38], in a manner independent of ubiquitin and ATP, whereas 26S removes, in a personalized manner, polyubiquitinated proteins [39,40,41].

In order to offer an in-depth view, previous work attributed to 26S revealed that it is highly susceptible to oxidative stress, resulting in its inactivation [42,43]. However, the reactivity is not detrimental, since it has been demonstrated to boost the 20S aiming, to clear irreparably damaged proteins [40,44,45]. On the other hand, 26S inhibition has another hazardous drawback, characterized by the accumulation of undegraded polyubiquitinated proteins being detected by the ubiquitinbinding of a unique enzyme having specific structural and functional features known as histone deacetylase 6 (HDAC6) [46].

Moreover, HDAC6 mediates this proteotoxic stress, which acts as a signal. In this way, a unique circuit is established as follows: (I) preventing the formation of heat shock proteins (hsps), (II) pro-inflammatory reactions induced the heme oxygenase-1 (HO-1) and nuclear factor erythroid 2-related factor 2 (Nrf2), (III) removal of polyubiquitinated structures by HDAC6, and, finally, (IV) lowering the proteotoxic stress by modulating the apoptosis through hsps and HO-1 [46,47,48].

Given that 2% of lipofuscin’s structure comprises metals [49], being especially abundant in catalytic iron, it appears to play a role in further oxidation reactions. To our surprise, after a considerable amount of time, a detailed intracellular activity of lipofuscin is still lacking, all remaining at a theoretical stage. Fortunately, Höhn and Grune [26] argued that lipofuscin is an important source of oxidants, concomitantly with the ability to incorporate iron in a redox-active manner.

Forward, it was demonstrated, a decade ago, by Höhn et al. [50], that artificial lipofuscin and iron-loaded artificial lipofuscin exacerbate the activity of caspase-3, being also used to investigate the viability of human fibroblasts [51]. Accordingly, the same authors postulated that inhibition of proteasome is responsible for the cytotoxic effect of lipofuscin, which is why macroautophagy rather “ensure: the uptake of lipofuscin into the lysosomes [52] in a manner dependent on the levels of oxidative stress [53].

To summarize the aforementioned, lipofuscin can be viewed as an aldehyde-linked and lipid remnant in a complex Schiff-base construct. Lipofuscin-loaded lysosomes still receive from the TGN substantial amounts of enzymes [54]. It seems that a macromolecular crosslinked reaction could be behind lipofuscin’s resistance to autophagy [11].

Lysosomes contain many important subcellular components, including mitochondria, where peroxisomes generate superoxides from xanthine oxidase [55]. They are not only subjected to autophagocytosis, but they also release hydrogen peroxide from the process of beta-oxidation of fatty acids, which can pierce the lysosomal membrane.

Superoxides act to reduce Fe(III) to Fe(II) in a Fenton-like reaction, which may further influence lipofuscin accumulation. In cases of stress or damage, there is a large influx of superoxides, above normal levels, into cells, which increases the production of specific radicals [56] and contributes to the exacerbation of lipofuscinogenesis.

Therefore, mitochondria and lysosomes are thought to create an important system that may even induce apoptosis [57]. Once lysosomal enzymes are released into the cytosol, the only factors known to induce programmed cell death are the pro-apoptotic sentinels’ Bid and Bax [58], the activation of phospholipase A2 (PLA2s) [59], caspases [60], 2-amino-4-trans-octadecene-1,3-diol (Sphingosine) [60], and lysosome-associated apoptosis-inducing protein (LAPF) [61].

In summary, mitochondria suffer structural alterations which lead to a reduction in adenosine triphosphate (ATP) synthesis [62], and they therefore increase the generation of free radicals [63]. This process disturbs the antioxidant system [64], which allows greater insult/damage to DNA [65], and disturbed apoptosis [66]. The lysosomal–mitochondrial axis is verified in this context. Long-lived aged cells have a decreased adaptability, and the proper turnover is impossible because they are fueled with lipofuscin [67].

## 3. Evolutive Matryoshka-Like Mechanism and Its Multifaceted Pattern

In this way, Hayflick and his colleagues were the first to demonstrate the limited proliferative ability of normal diploid cells because of telomere shortening [68,69]. They called this manifestation “cellular senescence”, being an antithetical phase of oncogenesis [70]. Accordingly, both clinical and instrumental diagnostic methods need to expand their current approaches [71] in order to efficiently counteract this abnormal attempt of the organism at sustaining youth.

Shelterin is a protein complex known to be the main defensive mechanism that prevents end-to-end chromosome fusions [72] that takes place with aging. Thus, reactive oxygen species (ROS) [73] produced during a lifespan could lead to a loss of potential of telomeric repeat-binding factor 1 and 2 (TERF1/2) [74].

Telomerase reverse transcriptase (TERT) is influenced by 8-Oxoguanine [74], whereas the ROS scavengers, such as N-acetylcysteine (NAC), delay any early senescence. This is why protection of telomere protein 1 (POT1) and telomeric repeat-binding factor 2 (TERF2), in this context, partially mediate p53 transactivation E3 ubiquitin ligase Sah1 [75] by blocking multiple aberrant DNA damage responses (DDRs) that could otherwise result in a premature senescence [76].

Telomere attrition during proliferation is preceded by a telomeric DNA loop destabilization and an uncapping of telomeres that lead to telomere-dysfunction-associated foci (TIFs) [77], or by a telomere-associated foci (TAFs) induced by oxidative damage at the level of telomeric G-reach repeats. It should be noted that this process is independent of length of the telomeres and whether or not they are under the guardianship of Shelterin [78,79].

Such an event is marked by an increased cell arrest, reflected by a defecatory ribosome biogenesis [80], as well as a reduction in the retrotransposons [81], especially after persistent DDRs. Additionally, DDRs promote a deposition of the H2A histone family member X (γH2AX) and p53-binding protein 1 (53BP1) [82] in chromatin.

Subsequently, it activates ataxia telangiectasia mutated (ATM), which is involved in the stabilization of p53 [83] and ataxia telangiectasia and Rad3-related (ATR), as an enhancer of the cell cycle checkpoints (checkpoint kinase 1/2—CHEK1/2) [77].

Nevertheless, senescence determines a chromatin reorganization, but the most pronounced change is the formation of senescence-associated heterochromatic foci (SAHFs) [84]. Alongside some nuclear substructures known as DNA segments with chromatin alterations reinforcing senescence (DNA-SCARS), which are different from the transient damage foci, SAHFs and DNA-SCARS constitute the broadest marker of cell senescence [85].

**Figure 1:**
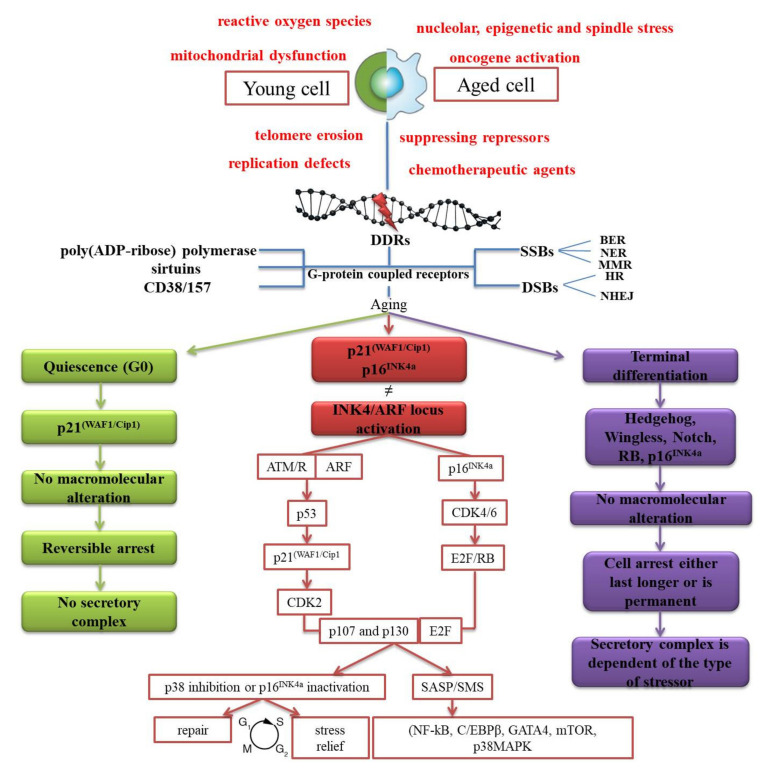
Distinct stressors which inflict alterations at the DNA level, during the lifespan, independently of the status of a cell (young or aged). Signals initiated in response are mediated by the G-protein coupled receptors. Subsequently, five highly specialized mechanisms are activated, and three specific NAD-consuming enzymes are used for re-establishing and maintaining the integrity of the human genome. Based on the type-related stressor, cells are subjected to a distinct type of withdrawal (red, green, or purple border).

Viewed initially as a static exit gate from the cell cycle via p21(WAF1/Cip1) [86] and p16INK4a [87], and a consequence of a finite ability of the cells to proliferate, cellular senescence proved to be a kinetic multistep process (Figure 1).

Senescence usually activates following exposure to hazardous intrinsic or extrinsic stressors [88] that manifests under single-strand breaks (SSBs) or double-strand breaks (DSBs). In both cases, base excision repair (BER), nucleotide excision repair (NER), and the mismatch repair (MMR) are undoubtedly the key guardians against SSBs [89]. On the other hand, homologous recombination (HR) and the non-homologous end-joining (NHEJ) are dedicated to repairing DSBs [90].

Insults detected at any level of a cell are mediated through G-protein coupled receptors [91]. They next transduce and transmit information to effectors, such as enzymes or ion channels [92]. Many cascades of signaling ensue by using seven-(pass)-transmembrane domain receptors to convert external and internal stimuli into intracellular responses.

The main classes of enzymes involved in initiating responses that revolve around the integrity of DNA, before and after a denaturation, are represented by three pillars of homeostasis, such as poly(ADP-ribose) polymerase, sirtuins (SIRT), and CD38/CD157. As ubiquitous metabolites [93,94,95], they participate in the maintenance of the balance between pro- and antioxidants [96], hinder any mitochondrial dysfunction [97], and act as a switch for the programmed cell death [98].

Moreover, p53-p21^CIP1^ and p16^INK4a^/pRb pathways (p107 and p130) [99] constitute the major effectors, with their whole functionality depending on INK4/ARF locus [100]. A series of cyclin-dependent kinases [101] phosphorylates RB family members and further represses the transcription factor E2F required for the cell-cycle progression [102].

It has been recently shown that product of INK4/ARF locus acts as a coating and sequesters the mouse double minute 2 homolog (MDM2), which contributes to increasing the levels of the protein p53 [103]; whilst forkhead box protein O4 (FOXO4) acts in the modulation of p53, localization, and transcription [104].

There are also cases when the cell arrest is only temporary and reinstated by appropriate stimuli via p27^Kip1^. When cells acquire a new phenotype and the withdrawal lasts longer, there is a series of volatile molecular pathways that are less explored and understood, such as Hedgehog, Wingless, Notch, RB, and p16^INK4A^ [105].

To proceed to the next phases, older cells must undergo a series of structural changes [106], to point where they come into contact with a plethora of cyto- and chemokines, matrix metalloproteinases (MMPs), growth modulators, and angiogenic factors [107]. However, there is also the possibility that such cells could persist for long intervals of time [108], to adapt and diversify [85], aside from the in vitro protocols [109].

The complex known as senescence-associated secretory phenotype (SASP) [110], or senescence messaging secretome (SMS) [111], is mediated by key translational factors such as the nuclear factor kappa-light-chain-enhancer of activated B cells (NF-κB), CCAAT/enhancer-binding protein beta (C/EBPβ), transcription factor GATA-4 (GATA4), mammalian target of rapamycin (mTOR), and p38MAPK signaling pathways.

It has been proposed that the sensing of cytoplasmic chromatic could be a trigger of SASP through the cGAS/STING pathway [112,113]. An additional layer of SASP was suggested to be mTOR, which controls interleukin’s 1 alpha (IL-1α) translation. mTOR directly mediates the SASP [114] and indirectly affects the butyrate response factor 1 (ZFP36L1) [115]. It activates the inflammasome and increases the reactivity of the cells under pathophysiological conditions [116]. This has the effect of concomitantly regulating biological aging [117,118], by triggering the immune system to eliminate the senescent components [106,119], through a reinforcement and spreading of the biological aging in an auto- and endocrine manner [107,111,120].

It can be concluded that the consequences of aging at the cellular level are a time-dependent through an abnormal synthesis of proteins, but also a genome instability [121]. Irrespective of number or type of cells, there are time-dependent alterations within genes expression [122] and mitochondrial potency [123], as well as the epigenome [124].

## 4. Is lipofuscin the Main Product of a Weak or Incomplete Recycling Activity?

Mechanically speaking, the balance between noxious and newly synthesised proteins is maintained by the ubiquitin-proteasome pathway (UPP) [125]. It functions at a cytosolic level, targeting proteins from cytosol exposed on the outer membrane of mitochondria and after retrograde transport from the endoplasmic reticulum [126,127,128]. At a macromolecular level, the balance is achieved by the autophagic mechanism programmed in eliminating worn-up/denatured structures [129].

Eukaryotic cells possess highly specialized organelles in the removal of all unnecessary biological structures [130]. However, lysosomes are not only mere terminal compartments for the processing of biological constituents, but also participate in a series of other biological processes such as modulation of nutrients availability [131], cell differentiation [132], apoptosis [133], and oxidative stress [134].

Their catabolic potential is wide, ranging from micro- to macromolecular components [135], which is ensured by a set of hydrolases [136], and a proton-pumping vacuolar-ATPase [137]. In addition, they represent a hub for mammalian target of rapamycin complex 1 (mTORC1) and transcription factor EB (TFEB). It acts as a master controller of autophagocytosis, being involved in its activation and as a biogenesis regulator [138].

Communication between these acidic vacuolar organelles and mTORC1 is granted by an ATP-sensing Na+ channel (lysoNaATP) [139] that blocks autophagosome biogenesis in cases of nutrient availability by phosphorylating the kinase complex ULK-1-ATG13-FIP200 [140]. Intriguingly, it supports longevity upon starvation [141] and exerts its activity at the level of the lysosomes surface [142], more precisely on amino acids.

Cell’s constant genesis depends on those parts that are removed and the use of basic fragments as building blocks for new assemblies [143]. As simple as it sounds at first glance, the mechanisms behind lysosomal degradation of old cells are closely correlated to autophagy(s) variants [144].

Autonomous to micro-, macro-, and chaperone-mediated autophagy that are discussed in the following, short-lived cytosolic proteins are decomposed and digested by calpains [145] and proteasomes [146]. Mitochondria possess their own proteolytic system, matrix Lon, and membrane-bound AAA proteases [147], where the structures are degraded without being subjected to autophagocytosis.

Another alternative autophagic route is represented by mitophagy, which focuses mainly on damaged mitochondria degradation [148], conferring cytoprotection against pro-apoptotic factors and ROS [149]. Lipophagy mediates energetic reserve [150], whose dysfunction is linked to metabolic deficiencies [151], whilst heterophagy degrades extracellular structures through endocytosis [152].

Autophagocytosis can be divided into three subtypes: micro-, macro-, and chaperone-mediated autophagy [150]. Chaperone-mediated autophagy (CMA) is characterized by a selective activity, thanks to its cytosolic heat shock proteins 70 (hsp70) and co-chaperones that recognize motif lysine–phenylalanine–glutamate–arginine–glutamine (KFERQ) and lysosome-associated protein type 2A (LAMP-2A) for particular proteins [153]. In the second form, microautophagy involves the invagination of macromolecules that are directly delivered to the lysosomes [154]. In the last form, macroautophagy is the most important way of removing large organelles, being highly conserved across different species [155]. The material is first embedded in a double-membrane vacuole (autophagosomes) and then transported to the lysosomes. At an early stage, autophagosomes fuse with the lysosomes, forming a single-membrane vesicle (phagophore) which contains useless material [156]. In particular, lysosomes that results from late endosomes are deprived of mannose 6-phosphate receptors, their pH is lower, and they also receive vesicles that carry hydrolases from the trans-Golgi network (TGN) [157].

With the discovery of the ATG family [158], our understanding of the steps involved in autophagocytosis recycling has also been improved, and it includes adjustment/origination, phagophore assembly and cargo loading/decomposition [159]. Any knockdown of isoforms like ULK1 or Beclin-1 result in a double-negative effect and severely reduce lifespan by accelerating age-related diseases [156].

Based on the aforementioned, the process of autophagy is considered to be multifaceted and this idea is highlighted by the cargo loading/decomposition stage which is connected to lysosomal activity. This stage is further divided into three distinct sub-stages: (I) reconnaissance and loading, (II) delivery to and merging with organelles, and finally (III) decomposition [160].

## 5. Oxidative Stress and the Exogenous Supply, Longevity Switchers?

Harman’s “free radical theory of aging” [161], which was later renamed “the oxidative stress theory” [162] explains some of the downfalls of being a strictly aerobic organism. For example, the higher the metabolic rate, the higher the ROS production. Even though oxidative stress (OS) is not an essential factor needed for aging [163], it has certainly been found to contribute to this process.

Other theories suggest that an exogenous supply of vitamins can re-establish this circuit through upregulation of the intracellular redox state and mitochondrial expenditure. This conclusion was issued following a series of in vivo studies conducted, targeting nicotinamide adenine dinucleotide (NAD+) levels in human patients [164], experimental models [165,166,167], and *Drosophila melanogaster* [168].

At present, NAD+ remains an obscure cofactor that has not been properly explored. Initially, it was believed to be involved in the modulation of cellular energy, but recent studies highlighted that NAD+ is a primary factor with a wide range of functions [169].

NAD+ is less commonly than NADH and is found at levels of 0.2–0.5 mM intracellularly, with a low pK_a_ value and its pyridinium is redox-active which predisposes it to substitution at the ribose carbone of the pyridine C-N bond under normal conditions. Therefore, NAD+ acts as a controller of catabolic and biosynthetic pathways [169,170], including oxidation of fatty/amino acids, oxidative phosphorylation, tricarboxylic acid (TCA) cycle, glycolysis, and pyruvate dehydrogenase pathways [171]. The level of NAD+ fluctuates in response to energetic stress [172], which simultaneously upregulates the production of ROS. On the other hand, NADP plays a role in early antioxidant defense, but, in some tissues, it could serve as a promoter of reactions that ultimately lead to the accumulation of free radicals [173].

Both NAD+ and NADP act as electron carriers in redox reactions of cells, and their reduced forms (NADH/NADPH) receive electrons by adding a hydride ion (in position four of the pyridine ring); over two hundred enzymes are necessary to reverse their reductive effects, such as in oxidation [173,174].

Following the transfer of a phosphate group from the ATP onto 2′-hydroxyl on adenosine ribose, the resulting phosphorylated precursor [175,176] is reduced [175] and is involved in all oxidation/reduction reactions in cells. NAD+ and NADH are involved in catabolic reactions and obtained using GAPDH (glyceraldehyde-3-phosphate dehydrogenase), which is a process that is opposed to the GNG (gluconeogenesis). NADP and NADPH ensure anabolic reactions, which are usually in excess, with a ratio of more than 1 compared with NAD/NADH [177].

Once human cytosolic NADK had been discovered, almost another ten years passed before mitochondrial NADK was unveiled. NADK homologous genes can be found in the genome of a variety of pathogenic entities [175], with the exception of *Chlamydia trachomatis*, [178,179,180]. In this way, there is a hint towards the underlying mechanism of NADPH production [180], which is largely dependent on the oxidative branch of the PPP (pentose-phosphate pathway) and metabolism of carbon atoms [181].

At the mitochondrial level, NADH is obtained via three steps during the TCA cycle, where acetyl-CoA is oxidized, to produce CO_2_. Thereafter, NADH is oxidized, using oxidative phosphorylation to produce energy, which is essential to the cells [182]. The intracellular ratio of NAD+ to NADH is usually 10:1, which further reflects its powerful catabolic role as an oxidant and as a metabolic reader of parameters [183].

Given the fact that 90% of all oxygen (O_2_) is taken up by our cells, mitochondria can be seen as the main producer of free radicals [184] through partial four-electron reduction of O_2_ to H_2_O [185]. Two distinct electron chain complexes were attributed to ATP production—complex 1 (NADH dehydrogenase) and complex III (ubiquinone-cytochrome *c* reductase) [186].

The most reliable way to reduce the effect of OS is to uncouple or reduce the metabolic rate [187]. By doing this, ATP would no longer be generated, and, instead, thermogenesis allows the production of heat using three distinct proteins (thermogenin-UCP1/2/3) [188]. However, the consumption of oxygen without ATP production will also lead to the formation of superoxide anions (O^−^_2_) [189].

However, OS does not always have an antagonist role and can play vital roles in the cell. For example, ROS produced from phagocytes or cytosol are crucial to combat infections or to control proliferative responses [190], by acting as an inflammatory signal-like molecules [191,192].

In such circumstances, NADPH acts as a substrate for NADPH oxidase in neutrocytes/ phagocytes and eliminates pathogenic entities by generating superoxide [193]. The lack of electron transfer from NADPH to cytochrome P450 promotes ROS production in the endoplasmic reticulum [194]. Since the discovery of all seven NADPH oxidase (NOXs) members, which were initially thought to produce ROS only in phagocytes, we have found that they are not limited to phagosomes, but rather fulfill functions such as free radical production [195] and signaling [196].

The major regulatory and vital processes in aging are represented by specific pro-inflammatory cytokines and enzymes involved in the immune system response and prostanoids synthesis [197]. However, it must be considered that cyclooxygenase (COX) is the predominant enzyme of prostaglandin (PGs) pathways [198]. PGs produce most of the reactive species during the conversion of prostaglandin G_2_ (PGG_2_) to prostaglandin H2 (PGH_2_) from arachidonic acid [199,200].

Unfortunately, we have not identified the literature studies that highlight a clear link between OS and lipofuscin. As a preliminary conclusion, an exacerbated ROS production certainly promotes an elevated lipofuscin accumulation in RPE cells, which are, as discussed, the most susceptible. This is possible since oxidative stress is a universal substrate to an impairment of the internal environment conditions, while lipofuscin is a residual product. Even so, this does not necessarily mean that they are not really interconnected.

## 6. It Is a “Gut” Feeling and Should Not Be Ignored

Metagenomic studies of the broader implications of intestinal microflora, considered the “second brain”, uncovered many of their secrets. Here, we talk strictly about those structures which form the enteric nervous system (ENS) [201,202,203,204].

From what it is known, key roles fulfilled by the enteric microflora include the following: (I) antimicrobial compound synthesis in order to block pathogen overgrowth, (II) secretion of immunoglobulin A (IgA) for the fortification of the intestinal epithelium, (III) nutrient absorption, and, finally, (IV) maturation of the immune system [205].

Inappropriate removal of these biological structures can again affect the body by triggering chronic low-grade and systemic inflammatory reactions (termed inflammaging), as these stressors promote the breakdown of tight junctions. Essentially, a penetration of intestinal epithelium may lead to endotoxins, which are synthesized by pathogens, circulating to the brain and stimulating a return deficient response, in this case, inflammatory reactions [206].

The intestinal lumen–blood barrier is the main guardian that ensures symbiosis, and alongside commensal microorganisms, it prevents the adherence of pathogens to the intestinal wall by protecting intestinal epithelial cells (IECs) [207], while Paneth cells may allow possible disruption of intestinal epithelium [208]. IECs are protected on the plasma surface by a series of glycoproteins, enterocytes, and gut-associated lymphoid tissue (GALT) which perform similar functions [209].

The goblet cells of the small intestine secrete mucin and mucin 2 (MUC2) [210,211], alongside antimicrobial peptides (AMPs), which strengthen the barrier against any pathogenic entity [212]. Some goblet-derived products such as trefoil factor 3 (TFF3) and Resistin-like molecule-β (RELMβ) form a physical barrier in response to inflammatory reactions, and RELMβ assists MUC2 in the regulation of adaptive lymphocytes and macrophages [213].

As mentioned above, there are enterocytes that are directly involved in host-defense that possess some pattern-recognition receptors (PRRs) for the detection of harmful species. These sensors contain toll-like receptors (TLRs) and nucleotide oligomerization domain-like receptors (NODs). In return, NODs perform an action similar to those of enterocytes, by targeting some microbial templates known as microbe-associated molecular patterns (MAMPs). This, in turn, activates NF-κB, NOD, and, subsequently, damage-associated molecular patterns (DAMPs) [214].

However, a dysbacteriosis will inevitably occur, and this is the case when all pathogens are engulfed and as antigen-presenting cells (APCs) are directed to dendritic cells (DCs) [214]. Microfolding cells present the antigen to naïve clusters of differentiation 4 cells (CD4) and allow differentiation into T helper cells (Th cells) and production of IgA [215].

In addition, we can also find conserved motifs known as pathogen-associated molecular PRRs amongst the gut flora which present on the surface of pathogens, and a “leaky gut” has the potential to exacerbate ROS production. Bacterial lipopolysaccharides (LPS) and other toxins are recognized by these PRRs and initiate downstream signals by activating NF-*κ*B [216], protecting the blood–brain barrier (BBB) against harmful products of metabolism [217].

The latest evidences suggest that many colonies of bacteria that populate our intestines have defined roles in food processing [218] and metabolic potency [219]. The fluctuations of gut colonies are the most conclusive and under normal circumstances have a pivotal role in host eubiosis [220]. This is highlighted by a wide range of metabolites [221,222], especially short-chain fatty acids (SCFAs). These metabolites result from fermentation of fiber and carbohydrates, but more specifically, acetate, butyrate, and propionate, which contribute to energy production for enterocytes [223]. They contribute to the optimal functionality of the neurohormonal axes [224] and act against age-induced disorders [225,226].

The hypothalamic–pituitary–adrenal (HPA) axis, which constitutes the core of stress-related mechanisms, was left behind [227]. Unfortunately, there has been little acceptance regarding the modulatory effect of oxytocin, or cortisol [228]. It has been recently demonstrated that patients diagnosed with major depressive disorder (MDD) had fluctuating levels of oxytocin and cortisol fluctuates [229,230].

Studies conducted with the aim of both expanding our knowledge of gut microflora implications and possible therapy are summarized in Table 1.

This could be the result of impaired processes along the HPA axis and may be associated with the first phase of some gastrointestinal disorders such as irritable bowel syndrome (IBS) [216]. As an untreated condition, the predisposition towards IBD is unquestionably higher. With aging, although it is not a mandatory criterion, the prevalence of metabolic disorders is accelerated, and, in the last phase, there is a prevalence towards neurodegenerative or psychiatric disorders.

Even though it is an under-evaluated topic, it certain is that gut microflora deeply manipulate human development and constitutes the main boundary between homeostasis and dysbiosis. More precisely, it is about the only relevant study identified in the current literature. Komura et al. [240] demonstrated, in 2013, that supplementation with *Bifidobacterium infantis* and *Caenorhabditis elegans* reduced lipofuscin accumulation by also improving locomotor activity and boosting longevity. Another powerful tool is recreating gut microflora in fecal transplantation. In this case, stool samples from long-living people were transferred to mice. Through this approach, mice had greater α diversity, especially in beneficial microorganisms such as Lactobacillus and Bifidobacterium, but also of short-chain fatty acid (SCFA)-producing entities (*Roseburia*, *Faecalibacterium*, *Ruminococcus*, and *Coprococcus*), as compared to the control [241].

## 7. Conclusions

Based on this report, it can be concluded that lipofuscin influences the optimum functionality of recycling homeostatic physiology. It is negatively associated with longevity, and it is implicated in various diseases, but at the same time, fragments of mitochondria that have not been completely degraded accumulate within lysosomes and promote abnormal longevity of aged cells by exacerbating ROS production. Besides ROS generation and irrespective of the status of organism, many other hazardous stressors promote the accumulation of insults at the DNA level that subsequently influence the cell cycle. These stressors are age-dependent, and they perpetually accumulate, invoking a rupture of the intestinal epithelium and accelerating other host systems and organs, which could ultimately result in the development of a neurological disorder, due to a disturbance along the gut–brain axis (GBA) and HPA axes.

## Figures and Tables

**Table 1:** Influence of the gut microflora upon defined phenotypical attributes.

Murine Model	Attribute	Procedure	Main Observations	Reference
Wistar rats	Anxiety-like behavior	Oral administration of a formula—Probiotic which contained *Lactobacillus helveticus* R0052 and *Bifidobacterium longum* R0175	Daily subchronic doses for fourteen days has significantly reduced anxiety in rats	[231]
BALB/c mice	Anxiety-like behavior	Daily administration of *Bifidobacterium longum* 1714 *Bifidobacterium breve* 1205, Escitalopram or vehicle	After one month and a half of treatment, both *Bifidobacterium* species and Escitalopram reduced anxiety, with no significant differences in corticosterone levels between groups	[232]
Wistar rats	Anxiety-like behavior	Administration of a mixture of *Lactobacillus acidophilus*, *Bifidobacterium lactis*, and *Lactobacillus fermentum*	After fourteen days of treatment, the cognitive activity of stressed rats was similar to that of controls	[233]
BALB/c mice	Depression-like behavior	Oral administration of *Lactobacillus rhamnosus* (JB-1)	Treatment with *Lactobacillus rhamnosus* (JB-1) diminished stress/anxious/depressive-related behaviors.	[234]
Sprague-Dawley rats	Depression-like behavior	Oral administration of *Bifidobacterium infantis*	Treatment with *Bifidobacterium infantis* has an anti-depressant activity, by restoring the HPA axis function, concomitantly with a pro-inflammatory cascade decline decrease	[235]
C57BL/6 mice	Depression-like behavior	Oral administration of *Lactobacillus helveticus* R0052, *Lactobacillus plantarum* R1012 and *Bifidobacterium longum* R0175, fluoxetine, or saline	Probiotic administration has diminished chronic mild stress by improving the overall immunity, followed by a significantly increase of *Lactobacillus* ratio	[236]
BALC/c and Swiss Webster (SW)	Depression-like behavior	Oral administration of fluoxetine or *Lactobacillus rhamnosus* JB-1	BALB/c mice displayed a significantly antidepressant-like behavior, while SW mice did not respond to treatment	[237]
Sprague-Dawley rats	Depression-like behavior	Oral gavage of *Bifidobacterium bifidum W37*, *Lactobacillus brevis W63*, *Lactobacillus casei W56*, *Lactobacillus salivarius W24*, *Lactococcus lactis W19* and *58* or vehicle	After one month and one week, treatment significantly reduced the major depression disorder by boosting both the immune system activity and tryptophan metabolism	[238]
Maternal immune activation mouse model	Stereotypic behaviors and vocalizations	Oral administration of *Bacteroides fragilis* or vehicle	Restores the social behavior after treatment; every two days for six days at weaning	[239]
